# Hybrid BO-XGBoost and BO-RF Models for the Strength Prediction of Self-Compacting Mortars with Parametric Analysis

**DOI:** 10.3390/ma16124366

**Published:** 2023-06-13

**Authors:** Asif Ahmed, Wei Song, Yumeng Zhang, M. Aminul Haque, Xian Liu

**Affiliations:** 1 Department of Geotechnical Engineering, College of Civil Engineering, Tongji University, Shanghai 200092, China; asifahmedbd@tongji.edu.cn (A.A.);; 2 Department of Civil, Construction and Environmental Engineering, The University of Alabama, Tuscaloosa, AL 35487, USA; wsong@eng.ua.edu; 3 Department of Civil and Environmental Engineering, The Hong Kong Polytechnic University, Kowloon, Hong Kong SAR, China; aminul.haque@polyu.edu.hk

**Keywords:** Bayesian optimization method, extreme gradient boost, mortar, strength prediction, random forest, self-compacting mortar, hybrid ML

## Abstract

Self-compacting mortar (SCM) has superior workability and long-term durable performance compared to traditional mortar. The strength of SCM, including both its compressive and flexural strengths, is a crucial property that is determined by appropriate curing conditions and mix design parameters. In the context of materials science, predicting the strength of SCM is challenging because of multiple influencing factors. This study employed machine learning techniques to establish SCM strength prediction models. Based on ten different input parameters, the strength of SCM specimens were predicted using two different types of hybrid machine learning (HML) models, namely Extreme Gradient Boosting (XGBoost) and the Random Forest (RF) algorithm. HML models were trained and tested by experimental data from 320 test specimens. In addition, the Bayesian optimization method was utilized to fine tune the hyperparameters of the employed algorithms, and cross-validation was employed to partition the database into multiple folds for a more thorough exploration of the hyperparameter space while providing a more accurate assessment of the model’s predictive power. The results show that both HML models can successfully predict the SCM strength values with high accuracy, and the Bo-XGB model demonstrated higher accuracy (R^2^ = 0.96 for training and R^2^ = 0.91 for testing phases) for predicting flexural strength with low error. In terms of compressive strength prediction, the employed BO-RF model performed very well, with R^2^ = 0.96 for train and R^2^ = 0.88 testing stages with minor errors. Moreover, the SHAP algorithm, permutation importance and leave-one-out importance score were used for sensitivity analysis to explain the prediction process and interpret the governing input variable parameters of the proposed HML models. Finally, the outcomes of this study might be applied to guide the future mix design of SCM specimens.

## 1. Introduction

In recent years, SCM has been widely used because of its novel properties. It is widely used in bricklaying [1], plastering [2], repair [3], and building decorations [4]. The technological, economic, and environmental benefits of SCM have been widely demonstrated [5,6,7,8,9,10]. The advantageous properties of SCM are its high fluidity [11] and simple placement in densely reinforced structures [12], which can minimize the requirement for skilled labor and the concrete casting period. As the old reinforced concrete structure deteriorates over time, it becomes necessary to repair the portions that suffer major damage. In such situations, SCM is essential as it offers significant advantages over regular mortars [13] and could be counted as an optimal replacement for restoring and rehabilitating reinforced concrete (RC) components [14]. Furthermore, SCM provides sufficient strength to maintain its shape, resist cracking, and ensure an adequate bond between the mortar and the reinforcement [15]. SCM also offers the advantage of easy repair due to its ease of application and mechanical benefits [16].

However, SCM strengths can be affected by various factors, such as the mix design, material types and quality, the temperature and humidity of the mixing and curing environment, and the construction method [17,18,19]. The relationships between these factors and the resulting strengths, e.g., compressive strength (CS) and flexural strength (FS), can be highly nonlinear and difficult to capture using conventional regression models [20,21]. More importantly, normal cementitious materials and SCM have distinct differences in their properties and performance. In terms of workability, normal cementitious materials require vibration to remove voids and achieve compaction, while SCM has a high level of fluidity and can self-compact without the need for vibration [22]. This property leads to lower water content and lower mixing energy for SCM during the mixing process. Additionally, normal cementitious materials typically require additional reinforcement, such as steel bars or fibers [23], to increase strength and prevent cracking, whereas self-compacting properties allow for a better distribution of fibers, reducing the need for additional reinforcement. SCM can also achieve higher strength due to better compaction and fiber distribution, while normal cementitious materials may have lower strength due to a lack of compaction and the need for additional reinforcement. Therefore, accurately predicting the strength of SCM is necessary to meet the concern. 

For the broad and successful applications of SCM, a robust model is necessary to predict SCM strength properties, where machine learning (ML) might be a promising option to achieve this goal. Nowadays, ML methods have been successfully developed to predict features such as the CS of cement composites [24,25]. Research has also been conducted to determine the characteristics of different kinds of concrete, including high-performance concrete [26], ultra-high performance concrete [27], self-healing concrete [28], and rubber-modified recycled aggregate concrete [29]. These studies have utilized a wide range of ML models, such as support vector machines [30] and random forests [31], to predict concrete compressive strength and flexural strength. 

Numerous ML models, such as AdaBoost [32], k-nearest neighbors [33], decision tree [34], support vector machine (SVM) [35], and extreme learning machine [36] were developed in previous studies to predict the compressive strength of traditional cement-based mortars. In addition, the bagging regressor algorithm was applied to examine how waste glass powder affects the flexural strength of cement mortar [37], and Artificial Neural Network (ANN) was utilized to predict the compressive strength of 3D-printed mortar [38]. A backpropagation (BP)-based neural network was used to predict mortar strength [39] and the strength of mortar with multiple admixtures [40]. Ly et al. (2022) [41] used the grid search to optimize the performance of the model and conducted sensitivity analyses of self-compacting concrete (SCC) through conventional formula. Other researchers such as Nguyen et al. (2022) [41] and Farooq et al. (2021) [42] achieved a higher accuracy with a comparatively large dataset along with sensitive analysis interpretation but did not specify any optimization method. 

For SCM, Khatibinia et al. (2016) [43] used 82 samples to predict the compressive and flexural strength through a wavelet–based weighted least squares–support vector machines (WLS–SVM) approach. The performance of ML models highly depends on the hyperparameter selection and the amount of training data. Such a small amount of training data makes it difficult to generate effective ML models. Additionally, the selection of the wavelet function and the number of decomposition levels can greatly affect the model’s ability to capture relevant features in the input data. The regularization parameter also plays a critical role in controlling the trade-off between model accuracy and complexity, which requires careful tuning to avoid the overfitting problem. However, no hyperparameter selection or tuning process was conducted, which could raise questions about the model’s predictive ability and possible overfitting problem. In 2022, Faraj et al. [44] used ANN to predict the compressive strength of SCM incorporating modified nano particles with a comparatively large amount of data, and achieved good accuracy. However, sensitivity analysis is not seen in the above studies. Thus, the most influential input parameters that affect the compressive strength of SCM remain unknown.

ANN has a high degree of flexibility in fitting data, which can result in an overfitting problem when the model is not properly regularized [45,46]. ANN is more prone to overfitting due to its flexibility when handling data, especially when the data is limited. On the other hand, Extreme Gradient Bost (XGBoost) has the ability to deal with outliers and provides regularization techniques, for instance L1 and L2 regularization, which can help prevent overfitting to noisy outlying data points. Zhou et al. [47] conducted an ML analysis predicting the comparative density of Ti-6Al-4V components based on a small dataset and showed that optimized XGBoost outperformed ANN and SVR models. In comparison with other engineering fields, experiment-based material databases tend to be smaller in sample size and have greater variety [48,49]. Small datasets are prone to overfitting and require the use of an optimized hybrid ML model to deal with potential overfitting problems. To overcome the limitations and improve the model performance, this study proposed a hybrid ML model based on XGBoost and Random Forest (RF) algorithms to perform ML analysis. In addition, the hyperparameters of this model are also fine-tuned via Bayesian optimization to achieve optimal prediction performance. Finally, cross-validation has been applied to assess the effectiveness of the proposed model to avoid overfitting and to obtain a more accurate prediction of unseen data.

In this study, we conducted an experiment on sand, binder, water, and their corresponding ratio to understand the strength behavior of SCM. The experimental specimens conducted in this study provided 320 data points for analysis. Two Bayesian optimized models, namely BO-XGB and BO-RF, were developed to predict the strength of SCM and to find the influential parameters through sensitivity analysis by employing the Shapley additive explanations. Additionally, the permutation importance and leave-out-importance methods were also used to perform the parametric analysis of the models and validate the results obtained from the shapely values. Finally, the efficiency of the models was also compared with traditional models using the uncertainly analysis.

## 2. Experimental Details

To make the database reliable and explicable, the authors produced SCM and performed strength tests independently. Cement, sand, water, and Meflux 2651F superplasticizer were employed to make the SCM. Three factors were varied in the experiment: the mass ratio of sand/binder, the mass ratio of superplasticizer/binder, and the curing days. The mass ratio of water/cement was set at 0.5. The water/cement proportion remained at a constant value of 0.5 for our experiment, since this is the minimum ratio required to make the cement mortar workable [50]. Experimental factors are presented in Table 1. The sand/binder ratio varies from 1.5 to 3.0. More emphasis was put on the conditions where the sand/binder equaled 1.5 and 2.0 for binder saving. When the sand/binder equaled 2.0, superplasticizer was added in ascending order from 0.10% to 0.23% until the fresh mixture began to bleed water. For the group with a sand/binder ratio of 1.5, only three dosages were tested because good flowability was accompanied with water bleeding problems.

**Figure 1:**
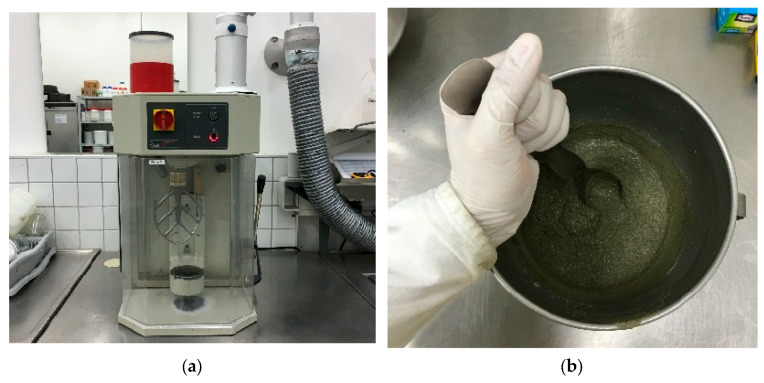
(**a**) stirring machine; (**b**) stirring by hand.

**Figure 2:**
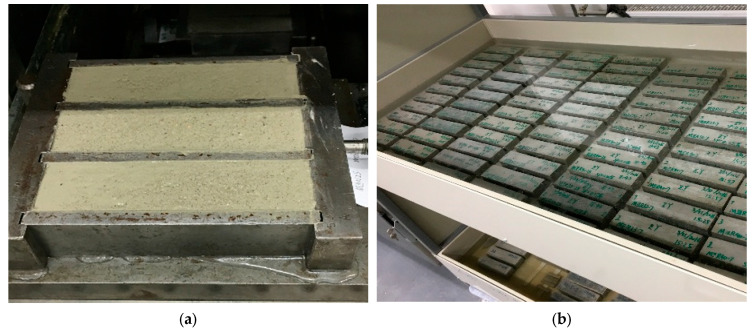
(**a**) SCM cast in the mold; (**b**) curing in the water pool.

To start with, the fresh mortar was made by both a stirring machine and hand visualized in Figure 1. First, the cement was weighed and placed into a pot. Second, water or water–superplasticizer solution was weighed and added, followed by lower-speed stirring for 30 s. During the mixing, water at room temperature was fixed at 20 °C. Then, sand was weighed and added, followed by high-speed stirring for 60 s. Next, 30 s of hand-stirring was performed, followed by a 60 s stand. After standing, 60 s of high-speed stirring followed, and the mixing procedure was complete. The fresh mortar was molded into a 40 mm × 40 mm × 160 mm specimen for curing. Curing was first performed in a curing box, as shown in Figure 2a. After 24 h ± 7.5 min, the specimen was unmolded and transported into a water pool for further curing (Figure 2b). During the curing, the water temperature was set at a constant 20 °C. The flexural tests and uniaxial compressive strength tests were conducted after 7 days or 28 days of curing.

**Figure 3:**
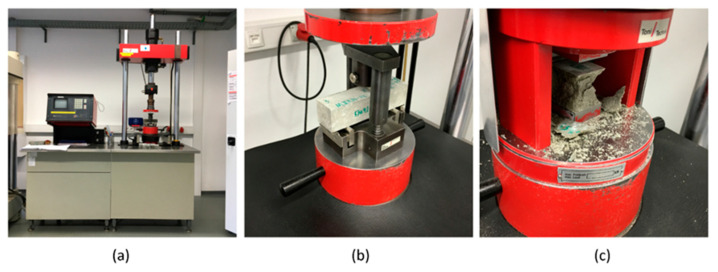
Experimental setup. (**a**) Toni technik compressive strength testing machine; (**b**) flexural testing procedure; (**c**) uniaxial compressive testing procedure.

The flexural tests and uniaxial compressive strength tests were performed according to the German code DIN EN 196-1 “Methods of testing cement–Part 1: Determination of strength” [51]. In the flexural test, the dimension of the tested specimen was measured first, including B, the width, and H, the height. Then, the specimen was placed on a Toni- technik compressive strength measurement machine visualized in Figure 3a. The length between the two support points L was 100 mm. Next, the loading speed was set to 0.05 kN/s, and the flexural test, visualized in Figure 3b, was performed until the failure of the specimen, where the force was recorded as F. The flexural strength was derived by the following equation:

(1) $$R_{f}=\frac{1.5FL}{BH^{2}}$$

After the flexural test, the specimen was split into two pieces, and a uniaxial compressive stress test was performed for each spilled piece. The uniaxial compression tests depicted in Figure 3c were also performed on the Toni technik compressive strength testing machine, but with a different fixture. The size of the loading surface was 40 mm × 40 mm. The loading speed was set to 2.40 kN/s, and the test was performed until the failure of the specimen, where the compressive force was recorded as C. The compressive strength was derived by the following equation:

(2) $$R_{c}=\frac{C}{40\times 40}$$

## 3. Database Development

**Figure 4:**
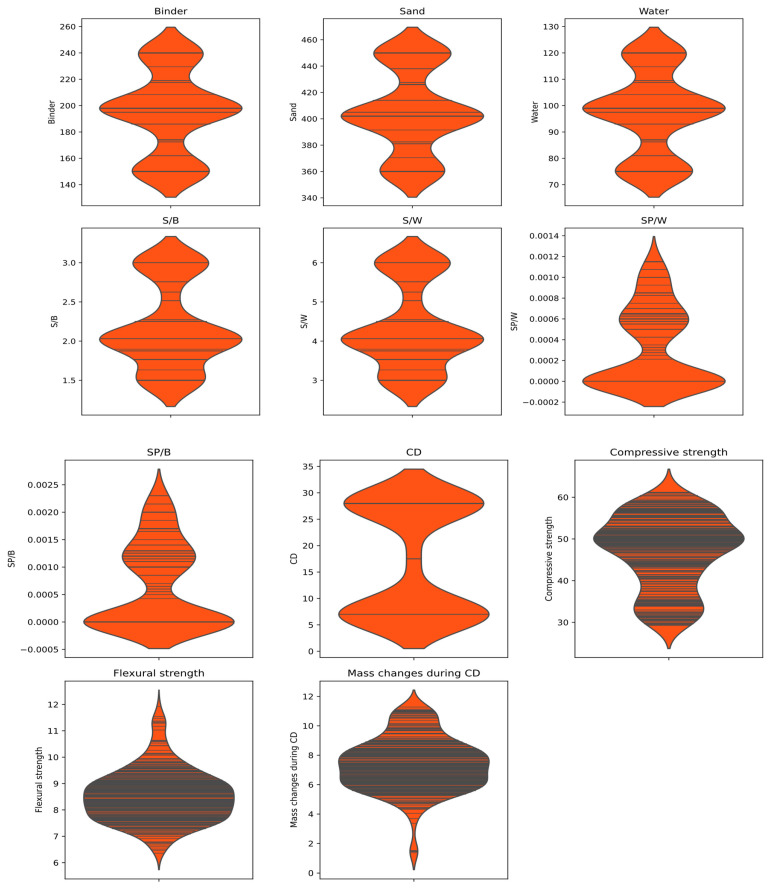
Swarm plot demonstrating the distribution of data points.

The building of ML prediction models largely depends on the construction of a reliable dataset. Therefore, a total of 320 data points were obtained from the experimental specimens and organized into a database to predict the strength of the SCM. The water-binder ratio was constant at 0.5 for all test samples utilized in this study. Machine learning models rely on data to identify patterns and relationships within that data [52]. To ensure our model can accurately predict the strength properties of SCM, we have expanded our database to include additional information on the sand, binder, water, superplasticizer, and their corresponding ratios. Nine input parameters are featured in our database to forecast the strength, and the input parameters are binder, sand, water, curing days (CD), sand–binder ratio (S/B), sand–water ratio (S/W), superplasticizer–water ratio (SP/W), superplasticizer–binder ratio (SP/W), and mass changes during CD. The two output parameters are flexural strength (MPa) and compressive strength (MPa). Figure 4 demonstrates the database in swarm plots. Each data point is plotted as a hexagon, and the density of hexagons in a particular region indicates the concentration of data at that value. The hexagons are arranged in a hexagonal grid, ensuring that the data points are spread out evenly and do not overlap, giving a clear representation of the distribution of the input and output variables. Table 2 depicts the statistical characteristics of the dataset’s inputs, output, and the performance characteristics of the model, detailed in terms of mean, standard deviation, minimum, and maximum values. Based on Table 2, it can be observed that 320 test samples were used with curing periods ranging from 7 to 28 days. The average amounts of sand, binder, and water used were 405.27 gm, 194.72 gm, and 97.36 gm, respectively. The sand–water ratio (S/W) did not exceed 6 gm, and the average sand–binder ratio (S/B) was 2.17 gm, with the maximum ratio being 3 gm. The average superplasticizer binder (SP/B) ratio was 594.81 gm, and the average superplasticizer water (SP/W) ratio was very low, at 0.00033 gm, to enhance workability. Our study found that compressive strength (CS) ranged between 0.054 MPa and 40.21 MPa, and flexural strength (FS) varied from 12.92 MPa to 601.11 MPa. 

**Figure 5:**
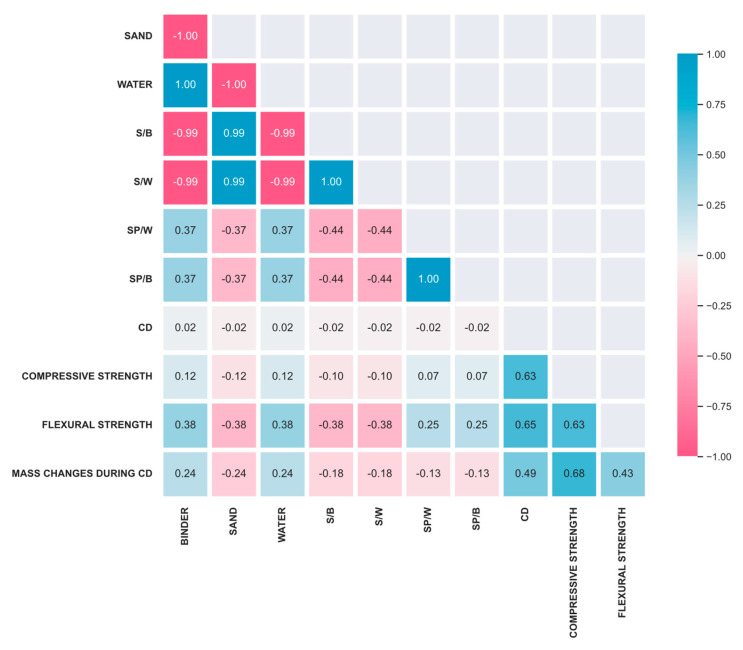
Correlation between input and output parameters.

Figure 5 presents a Pearson correlation matrix that demonstrates the correlation between the input and output variables. The Pearson correlation method is a commonly used approach to assess the strength and direction of the linear relationship between two numerical variables [53]. It assigns a value within the range of −1 to 1, where a value of 0 indicates no correlation, 1 signifies a perfect positive correlation, and −1 represents a perfect negative correlation [54]. The correlation between two input variables, X and Y, is the measure of the linear relationship between their attributes. The plot shape of the correlation matrix becomes symmetrically diagonal due to the two parameters being linked together in each square. If the correlation value is closer to zero, there is no linear trend between the two variables. A higher correlation value, closer to the positive one, indicates that the input parameters are more associated with each other, and if one parameter increases, the other also increases. Similar outcomes can be achieved when the correlation is closer to a negative value, but in this case, if one variable increases, the other decreases.

The analysis showed a slight difference in the correlation coefficients between CD and CD (0.63) when compared to CD and FS (0.65), indicating a slightly stronger correlation with FS. The correlation between the sand, water, and binder, as well as their corresponding ratios, exhibits a consistent positive and negative correlation, with a coefficient of 0.37. However, a significant negative correlation is observed between water and binder ratios and their association with sand, with a coefficient of 0.99. It is noteworthy to mention again that the water–binder ratio remained fixed throughout the experiment. Consequently, the ratio of S/B with S/W and SP/B with SP/W demonstrate a highly positive correlation, with a coefficient of 1. Other corresponding coefficients almost show the same trend.

The combined data preprocessing and normalization process is necessary because it brings all features in a dataset to a similar scale, ensuring that each feature contributes proportionally to the learning process. It improves model performance, prevents one feature from dominating others, and enables the optimization algorithms to work more efficiently. The mathematical expression is shown below

(3) $$X_{n}=0.1+0.8X\frac{X_{i}-X_{min}}{X_{min}-X_{max}}$$

Here, $X_{n}$ is defined as a normalized value of the variable, and $X_{i}$ is the original value of the variable. $X_{min}$ and $X_{max}$ are the minimum and maximum value of the variable in the dataset.

## 4. Employed ML Models

XGBoost and Random Forest (RF) models are popular machine learning algorithms known for their ability to handle complex, non-linear relationships in data and provide accurate predictions. They have been widely used in numerous fields, including materials science, and have demonstrated high performance compared to other traditional regression methods. Bayesian hyperparameter optimization is a powerful technique used to search the hyperparameter space of machine learning models to search the optimal combination of hyperparameters that yield the best performance. It can increase the accuracy and efficiency of the models by tuning the hyperparameters to the specific dataset and model architecture. 

In this study, XGBoost and RF models were chosen in this study due to their proven performance and ability to handle complex data, while Bayesian hyperparameter optimization was employed along with cross-validation techniques to enhance the models’ accuracy. This hybrid approach aimed to optimize the models’ parameters and improve their overall performance.

### 4.1. Extreme Gradient Boost (XGBoost)

The XGBoost algorithm is a well-known technique in the gradient boosting category, which performs admirably for classification and regression tasks. The approach relies on the idea of “Parallel boosting,” which creates a powerful learner by combining additive training methods with the prediction of weak learners. To prevent overfitting and maintain optimal processing performance, XGBoost is indispensable for prediction and regularization incorporation while minimizing objective functions. In practical applications, it has the potential to serve as a versatile computing library that blends different algorithms with the decision tree approach, resulting in improved estimation precision.

Let us assume a data set $\left\{m=\left(x_{1},y_{1}\right)\ldots \left(x_{l},y_{l}\right)\right\},where|l=1\ldots n$. The input data set is a custom-designed matrix and specified vector outcome, which has been used for training. The proposed tree ensemble model utilizes n adaptive functions to accurately estimate system response. By combining multiple decision trees, it can capture complex input–output relationships.

The input data set $x\in R^{m\times n}$ is a custom-designed matrix, and $y\in R^{l}$ is our specified vector outcome, which has been used to training the data set. The proposed tree ensemble model utilizes *n* adaptive functions to accurately estimate system response. By combining multiple decision trees, it can capture complex input–output relationships.

(4) $$\overset{}{{\overset{⌒}{y}}_{l}}=\phi \left(x_{l}\right)=\overset{n}{\underset{n=1}{\sum }}f_{n}\left(x_{l}\right),f_{n}\in T$$

Here, *T* denotes the space of the regression trees, which can be described as follows:

(5) $$T=\left\{f\left(x\right)=A_{t\left(x\right)}\right\}\left(t:R^{l}\to Z,A\in R^{m}\right)$$

The symbol *T* represents the tree structures, while *Z* and *A* represent the number of leaf nodes and their respective weights. Additionally, $f_{n}$ is represented as a function that combines *A* and *T* corresponding to the independent tree.

To optimize the ensemble tree and minimize errors, The OA of the XGBoost function can be minimized using the following approach.

(6) $$y^{z}=\overset{n}{\underset{l=1}{\sum }}h\left(y_{l},{\overset{⌒}{y}}_{l}{}^{\left(t+1\right)}\right)+f_{l}\left(x_{l}\right)+Q\left(f_{t}\right)$$

where $y_{l}$ is considered a quantifiable metric, ${\overset{⌒}{y}}_{l}$ represents a predicted value and *h A*. convex function, also known as a loss function, which is used to measure the exact and estimated values. The number of times the model iterates is denoted as *t* to reduce errors, and *Q* represents the penalty imposed for the complexity of the regression tree model.

(7) $$Q=y^{z}+\frac{1}{2}r||B|{|}^{2}$$

Here, B stands for the total vector score counted in the leaves, r regulates the regularization parameters, and y computes the lowest loss necessary to divide the leaf node of the above function. 

### 4.2. Random Forest (RF)

The Random Forest Regression (RF) model is a powerful technique that utilizes an ensemble of decision trees with bootstrapping and aggregation methods to optimize decision tree algorithms. This approach uses a random subset of predictor variables to construct each tree, resulting in a more diverse set of models. Furthermore, each tree is created using bootstrapped samples, ensuring that the model is robust and resistant to overfitting.

Let us presume to construct d trees with a total length of $k_{d}\left(x\right)$. The equation shown below yields the RF regression prediction model.

(8) $$U\left(x\right)=\frac{\sum_{l=1}^{d}K_{d}\left(x\right)}{d}$$

RF regression employs an array to produce *K* outputs for each tree from a collection of d trees. Here, $\left\{k_{1}\left(x\right),k_{2}\left(x\right),\ldots k_{d}\left(x\right)\right\}$, where $X=\left\{x_{1,}x_{2},\ldots ,x_{d}\right\}$ is a Y multi-dimensional input vector that produces a forest output *n*, which correspondent to each tress calculated as $\beta_{n}$ where n=1,2,…k. Every time a regression tree is generated, an updated training set, known as a bootstrap sample, is generated by drawing samples from the initial training set. This involves drawing an array of samples from the original training set in a randomized manner, while simultaneously replacing some of the samples.

During the process, a new regression tree is created in each iteration using a randomly selected training sample drawn from the initial data set. The accuracy of the regression outcome is then assessed using an out-of-bag (OOB) sample, which comprises the data points that were not selected for the training sample. To validate the accuracy of the measurements, the regression outcome is evaluated using two-thirds of the new training sample, while the remaining one-third of the OOB sample is used to check precision. This process is repeated for each regression tree, with a new randomized training sample being selected in each iteration. The following equation described the process numerically, where *JI* is the combines and *U* is the output array of the tree.

(9) $$JI\left(k_{d\left(xl\right)}\right)=1-\overset{n}{\underset{l=1}{\sum }}U{\left(k_{d\left(xl\right)},n\right)}^{2}$$

When using unused test data, validation features that are built into random forests improve the model’s ability to make accurate predictions when working with unfamiliar test data. The success of the random forest prediction model is determined by its ability to correctly estimate the outcomes of test samples that have not been seen before.

### 4.3. Bayesian Hyperparameter Optimization with Cross-Validated Evaluation

Hyperparameter tuning is a critical step in the machine learning workflow, as it can have a high impact on the performance of the model. Hyperparameters are parameters that are not learned from the data, but instead must be set manually before training the model. The process of finding the best hyperparameters involves trying out different values for each hyperparameter and selecting the combination that results in the best performance on the validation set. Manually tuning these hyperparameters can be challenging and time-consuming, as it requires a trial-and-error process of changing hyperparameters and retraining the model until the best performance is achieved [55]. This can be especially difficult when there are many hyperparameters, as the search space becomes very large and it can be difficult to determine which hyperparameters are most important to tune [56]. There are several techniques available for hyperparameter tuning in machine learning, including grid search, random search, Bayesian optimization, gradient-based optimization, and evolutionary algorithms [57]. Each technique has its own advantages and disadvantages, and the optimal method depends on the specific problem and dataset.

For this study, we used Bayesian hyperparameter optimization and the cross-validation method, which provides a more accurate prediction of the model’s generalization performance and helps to avoid overfitting [58]. Bayesian optimization with cross-validation can be more efficient than grid search and more effective than random search in finding the optimal hyperparameters for an ML model [59]. By using probabilistic modeling and Bayesian inference, the algorithm can guide the search toward promising hyperparameter values and converge to the optimal solution more quickly than the grid search optimization method [60].

**Figure 6:**
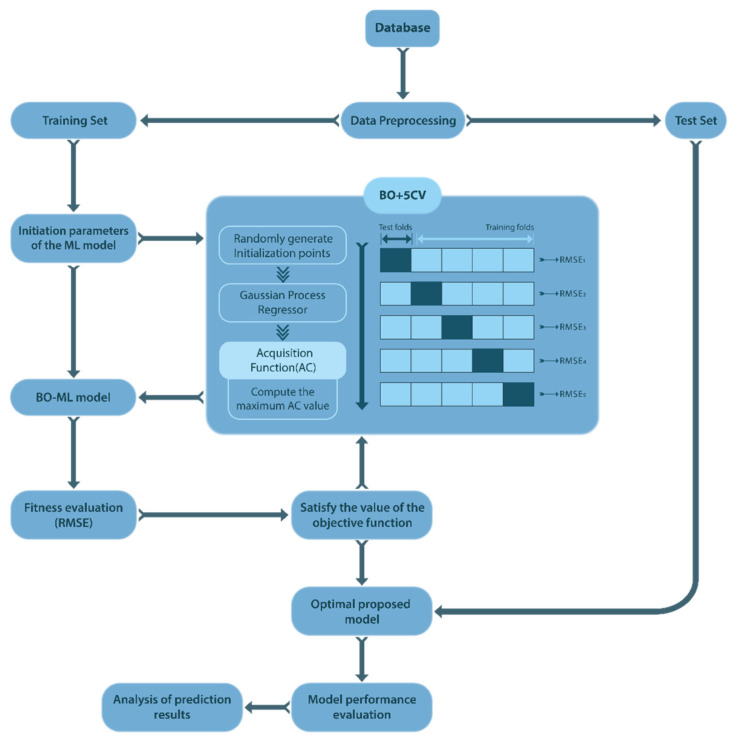
The framework of BO–ML model for this study.

For XGBoost and RF, a grid search can be computationally expensive and unproductive due to the large number of hyperparameters that need to be tuned [61]. Random search, on the other hand, can be more efficient in terms of computation time, but it may not find optimal hyperparameters due to its random nature [62]. The combination of Bayesian optimization and cross-validation provides a powerful and robust way to optimize machine-learning models and can significantly increase the efficiency and effectiveness of the model-tuning process. The detailed structure of our proposed model is illustrated in Figure 6.

In the Bayesian optimization process, it is necessary to choose a prior function to assume the distribution (for instance, a Gaussian process) of the objective function that is going to be optimized [63]. Therefore, the relationship between hyperparameters and model performance is assumed to be a Gaussian distribution function, where the average represents the expected performance, and the covariance function describes the correlation between different hyperparameter settings. The utility function that specifies subsequent evaluative steps is derived from the model’s posterior distribution via a Probability of Improvement (POI) acquisition function [64].

(10) $$PI\left(X\right)=\varphi \left(\frac{\mu \left(x\right)-f\left(x^{\prime }\right)-\varepsilon }{\sigma \left(x\right)}\right)$$

Here, $f\left(x^{\prime }\right)$ is the reported maximum value of the optimized function $f\left(x\right)$, $\mu \left(x\right)$ and $\sigma \left(x\right)$ are, respectively, the mean and standard deviation of the model at point *x*. The item ε is a minimal positive constant to encourage exploration of the target value. The goal of Bayesian optimization is to generate the best possible set of parameters by locating the global maximum or minimum value of the function *f*(*x*) in the number of possible values K.

(11) $$X^{*}=\arg_{x\in S}\max f\left(x\right)$$

In this study, the parameters used in the hybrid model are provided as input data for the function $f\left(x\right)$, and the output of the function is the maximum accuracy achieved by the model. The objective is to identify the optimal combination of parameters that produces the highest accuracy value. 

### 4.4. Model Assessment Criteria

In this study, ten statistical performance metrics were employed, specifically coefficient of determination (*R*^2^), mean squared error (*MSE*), root mean squared error (*RMSE*), mean absolute error (*MAE*), absolute percentage error (*MAPE*), objective function(*OBJ*), Scatter index(*SI*), Nash–Sutcliffe efficiency (*NSE*), Variance Accounted For (*VAF*), and Root Relative Squared Error (*RRSE*), which were used to measure the precision of the model. These metrics allow for a comprehensive evaluation of the model’s predictive ability by measuring its performance against the actual values in the dataset. By analyzing these metrics, the study aimed to assess the degree of accuracy and precision achieved by the model, thereby providing insight into its potential utility in predicting the shear behavior of SCM. The expressions of these statistical criteria are as follows:

(12) $$R^{2}=1-\frac{\sum_{l=1}^{n}{\left(y_{l}-\hat{y_{l}}\right)}^{2}}{\sum_{l=1}^{n}{\left(y_{l}-\bar{y}\right)}^{2}}$$

(13) $$MSE=\frac{1}{n}\overset{n}{\underset{l=1}{\sum }}{\left(y_{l}-\hat{y_{l}}\right)}^{2}$$

(14) $$RMSE=\sqrt{\frac{1}{n}\overset{n}{\underset{l=1}{\sum }}{\left(y_{l}-\hat{y_{l}}\right)}^{2}}$$

(15) $$MAE=\frac{1}{n}\overset{n}{\underset{l=1}{\sum }}\left|y_{l}-\hat{y_{l}}\right|$$

(16) $$MAPE=\frac{100\%}{n}\overset{n}{\underset{i=1}{\sum }}\left|\frac{y_{i}-\hat{y}}{y_{i}}\right|$$

(17) $$OBJ=\left(\frac{N_{tr}-N_{te}}{N_{all}}\right)\left(\frac{RMSE_{tr}+MAE_{tr}}{1+R_{tr}}\right)+\left(\frac{2N_{te}}{N_{all}}\right)\left(\frac{RMSE_{te}+MAE_{te}}{1+R_{te}}\right)$$

(18) $$SI=\frac{RMSE}{\bar{y}}$$

(19) $$NSE=1-\frac{\sum_{i=1}^{n}{\left(y_{l}-\hat{y_{l}}\right)}^{2}}{\sum_{i=1}^{n}\left(y_{i}-\bar{y}\right)}$$

(20) $$VAF=1-\frac{\mathrm{var}\left(y_{l}-\hat{y_{l}}\right)}{\mathrm{var}y_{i}}$$

(21) $$RRSE=\sqrt{\frac{\sum_{i=1}^{n}{\left(y_{l}-\hat{y_{l}}\right)}^{2}}{\sum_{i=1}^{n}{\left(y_{l}-\bar{y_{l}}\right)}^{2}}}$$

Here, $y_{l}$ denotes the true value or experimental value for both CS and FS. In contrast, $\hat{y}$ refers to the estimated value calculated by the machine learning model for CS and FS, whereas $\bar{y}$ denotes the arithmetic mean of y shear behavior values in the dataset. $N_{tr}$, $N_{te}$ and $N_{all}$ are the employed number of training, testing, and all data.

### 4.5. SHAP Analysis

In 2017, Lundberg et al. [65] proposed SHAP (Shapley Additive explanations) to evaluate the output of a model. SHAP estimation relies on game theory [66,67] and local explanation [68,69] to measure the contribution of each parameter in predicting the model’s output. Consider our BO-XGB and BO-RF model that predicts an output (N) from a set of N (for *n* features). The computation of Shapely values relies on a set of principles that ensure a fair distribution of the contribution of each feature, and each individual feature’s impact is weighted according to its impact on the model output, as shown below:

(22) $$\phi_{l}=\underset{S\in N\left\{l\right\}}{\sum }\frac{\left|S\right|\left(n-\left|S\right|-1\right)!}{n!}\left[v(S\cup \left\{l\right\}-v\left(S\right)\right]$$

Here, $\phi_{l}$ is the contribution of respective feature *l*. Variable *n* represents the total number of features, *S* is a subset of the features excluding *l*, and $v\left(S\right)$ represents the model output with only the subset of features *S*. The calculation of $\phi_{l}$ involves computing the difference in model output between including feature *l* in the subset *S* and excluding it, over all possible subsets of *S*. The resulting value represents the incremental impact of feature *l* to the overall model output. Based on the subsequent additive feature attribution technique, a linear function of binary characteristics *g* is established:

(23) $$g\left(z^{\prime }\right)=\varphi_{0}+\overset{n}{\underset{i=1}{\sum }}\varphi_{i}{z^{\prime }}_{l}$$

where, $z^{\prime }\in {\left\{0,1\right\}}^{n}$, and the binary vector *z* of length *n* is composed of elements that are either 0 or 1, depending on the presence or absence of a particular feature [65].

### 4.6. Permutation and Leave-One-Out Importance

The permutation importance (PI) is a method used to assess the relevance of every input variable on the predicted output. The method works by randomly shuffling the values of one feature in the dataset and measuring the model performance decrease. A feature that has a high impact on model performance will lead to a greater decrease in accuracy when its values are randomly shuffled. PI can be calculated for each feature and used to rank them according to their relative importance in the model. This technique is particularly useful in identifying which features have the greatest influence on the model’s outcome.

PI evaluates the relationship between a specific input parameter Xi and the predicted output Y while keeping all other input parameters constant. The PI score for a single input feature is calculated by measuring the difference between the MAE of the original model and the MAE of the randomly permuted input feature model. This approach determines the variability in the error calculation resulting from the input variable’s permutation. It is defined as the decrease in the model’s performance when a feature is randomly shuffled. The following equation expresses the relation explicitly:

(24) $$PI=MAE_{permuted}-MAE_{truth}$$

Here, $MAE_{permuted}$ is the *MAE* of the model when a variable’s values are randomly permuted, and $MAE_{truth}$ is the *MAE* of the original model with all input variables.

Leave-one-out (LOO) error is a statistical method that evaluates how well a learning algorithm performs. It provides a more accurate and unbiased assessment of the model’s performance compared to the empirical error. It is commonly used to choose the best model among a set of models. The leave-one-out error works by leaving out one observation from the training dataset and using the remaining dataset to train the model. The model is then tested on the left-out observation, and this process is repeated for each observation in the training set. The mathematical equation is described as

(25) $$LOO=\frac{1}{n}\overset{n}{\underset{i=1}{\sum }}L(y_{i},f\left(x_{i};X_{i}\right))$$

Here, *n* represents the total number of datapoints used for training the model, $x_{i}$ represents the individual datapoint in the dataset, $X_{i}$ represents the remaining data points after $x_{i}$ has been excluded, $f\left(x_{i};X_{i}\right)$ represents the predicted value of the model for $x_{i}$ using the remaining data points, *L* is the loss function, and $y_{i}$ represents the true label for $x_{i}$. The *LOO* method is helpful as the dataset in this study is small, and for each input parameter observation, it is important to know its significance in predicting the output parameter.

The integration of permutation importance and leave-one-out importance methods in sensitivity analysis provides a comprehensive understanding of the model’s feature importance. Permutation importance provides a global feature importance ranking, while leave-one-out importance evaluates the impact of individual features on model predictions. Additionally, the use of these methods can identify the most significant features and assess their contribution to model accuracy, which can be useful in deciding feature ranking. The integration of permutation and leave-one-out importance methods provides a robust sensitivity analysis framework for understanding the model’s feature importance. More detailed explanations can be found in these studies [70,71,72].

### 4.7. Uncertainty Analysis

Uncertainty analysis is a valuable technique used to assess and quantify the uncertainty associated with model prediction accuracy [73,74,75]. It involves the evaluation of input uncertainties and their propagation through the model to obtain output uncertainties. This study utilized an expanded uncertainty range to quantify the potential variation in the model with a 95% confidence level and provided a measure of the potential variation associated with the model prediction results. This approach allows for a robust interpretation of the results and facilitates decision-making based on the model’s uncertainty level.

(26) $$U_{95}=1.96\sqrt{\left(SD^{2}+RMSE^{2}\right)}$$

Here, *SD* represents the standard deviation of the model performance error, and 1.96 represents the critical value for a 95% confidence interval in a standard normal distribution. 

## 5. Results and Discussion

### 5.1. Hyperparameter Tuning

Hyperparameters regulate the functioning and architecture of the ML models’ training process. Creating an effective machine learning model requires a systematic and iterative approach that integrates algorithm selection and hyper-parameter tuning while considering computational efficiency [76]. After selecting an algorithm, the successive step is to fine-tune the model’s hyper-parameters to achieve optimal performance. This involves exploring different combinations of hyper-parameters and evaluating their impact on the model’s accuracy, overfitting, and computational efficiency. The objective is to identify the optimal configuration that maximizes the model’s accuracy while minimizing overfitting and computational cost.

It is common practice to split the dataset into training and testing groups to minimize the risk of model overfitting and improve its generalizability. In this study, the dataset was partitioned into a training set and a testing set using a 70/30 split, with 70% of the data being used for training the model and the remaining 30% reserved for testing its performance. In addition to the train–test split, the study also employed the five-fold cross-validation technique to further assess model performance. This approach involved dividing the data into five distinct subsets; the first subset used the testing purpose, while the remaining four subsets were used for training purposes. By repeating this process five times and evaluating the model’s performance at each iteration using the held-out subset, this study aimed to obtain a more reliable estimate of the model’s predictive power. Table 3 presents the most optimal parameter settings for the BO-RF and BO-XGB algorithms, which were determined by fine-tuning the model using our database.

The performance of these ML models is highly dependent on the hyper-parameter settings. As a result, this study utilized a Bayesian optimization method to repeatedly adjust these parameters and achieve better SCM data predictions. The hyper-parameter values used in the optimized models are presented in Table 3. It is evident that both models have similar parameter settings; thus, the accuracy difference is also not large.

### 5.2. Prediction Performance of Hybrid ML Models

This study used a comprehensive computational environment that employed well-known programming languages such as Python and its dependable ML packages, which are widely used in the machine learning industry. Furthermore, we conducted a detailed analysis of the model, focusing on the performance improvement achieved through hyperparameter tuning to combine our overall analysis. The BO-XGB and BO-RF algorithms were implemented using the Sklearn package on Python 3.9. Bayesian hyperparameter optimization was performed using the SKOPT package on Python. All calculations were performed on a laptop computer equipped with a 12th generation Intel Core (TM) i7-12700H processor with an Nvidia GeForce RTX 3060 Laptop graphical processing unit (GPU) and the Windows 11 operating system. Table 4 illustrates the assessment of the machine learning models’ precision based on multiple metrics, including the R^2^, MAE, MSE, RMSE, and MAPE. The satisfactory performance of a model is determined by the minimal difference in errors between its training and testing datasets [77]. Estimating metrics such as the R^2^, MAE, MSE, RMSE, and MAPE are employed to assess the effectiveness of each method. The R^2^ assesses the model’s goodness of fit, while MAE and MSE measure the average differences between predicted and actual values. RMSE and MAPE provide additional information on the models’ standard deviation and relative accuracy, respectively [78]. 

**Figure 7:**
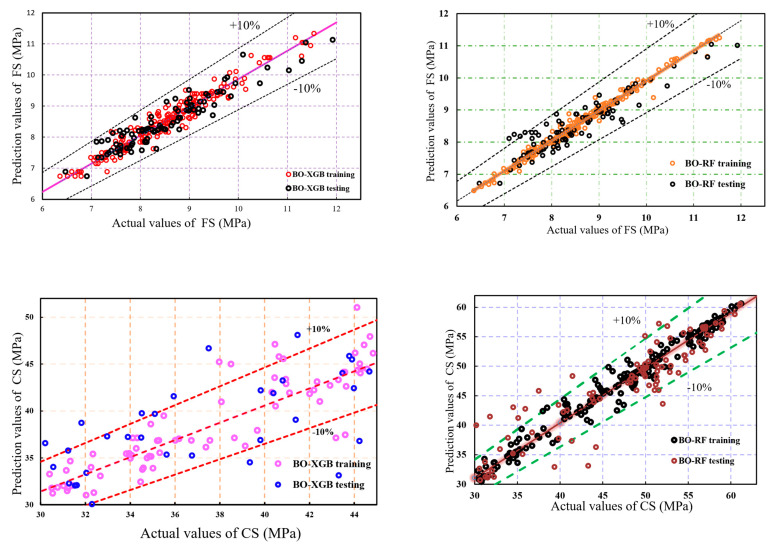
Comparison of actual and predicted values of the training dataset.

Figure 7 displays the scatter plot of the observed data against the simulated data for both the training and testing stages. The error range of the steep linear fit is indicated for both datasets, with the range being between −10% and +10%. Notably, the analysis reveals that over 87% of the simulated data samples of the two hybrid ML models were closely aligned with the linear fit. This observation implies that the proposed models were successful in predicting the shear behavior of SCM with a higher degree of accuracy than the traditional XGBoost and RF model.

By plotting the actual versus predicted values for each ML model, it can be observed that the BO-RF model shows a higher degree of correlation between the predicted and actual values compared to the BO-XGB model. Specifically, the data points for the BO-RF model are clustered closer to the line of perfect correlation, indicating a better fit between the predicted and actual values. On the other hand, the BO-XGB model appears to have more scattered data points, especially in predicting compressive strength, suggesting a lower degree of accuracy in predicting the actual values. Furthermore, the scatter plot in Figure 7 demonstrates that the predicted FS performance accuracy is better than that of CS for both the BO-XGB and BO-RF models. Specifically, it can be observed that the data points for the FS model are more closely clustered around the trend line compared to the CS model. This finding suggests that the FS strength model has a higher degree of accuracy in predicting the actual values compared to the CS model.

### 5.3. Prediction Performance of Hybrid ML Models

The models were trained on 70% of the dataset and tested on the remaining 30%. From Table 4, it can be seen that for the FS strength prediction in the training dataset, BO-XGB achieved a high coefficient of R^2^ of 0.96 and a MSE of 0.01, demonstrating a strong correlation between the predicted and actual values, where BO-RF had a lower R^2^ value of 0.95 and a similar MSE value with BO-XGB. Similarly, on the testing dataset, BO-XGB and BO-RF had R^2^ values of 0.91 and 0.90, respectively. BO-XGB outperformed BO-RF in terms of R^2^, MAE, and MAPE, where lower values indicate better accuracy. However, the MAE and RMSE values for both models were very similar, indicating that they had a similar level of accuracy in predicting FS. Interestingly, the BO-RF model had a slightly higher MAPE (0.03) compared to the BO-XGB model (0.02), indicating that it was more inconsistent in its predictions.

**Figure 8:**
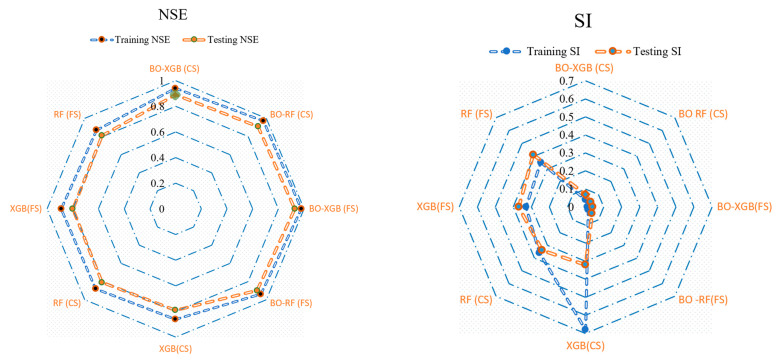
Assessing goodness of fit in hybrid ML models using radar diagrams.

The evaluation criteria of NSE and SI were used to assess the performance of two hybrid ML models, using both observed and simulated data points at the training and testing phase. The analysis results, presented in Figure 8 through the Radar plots [79], demonstrate that these statistical parameters can effectively measure the adjustment performance of the models to the data sets. Specifically, the two hybrid ML models showed significantly higher NSE and less SI values during both phases of the data set. These findings suggest that the evaluated hybrid ML models performed well in accurately modeling the data set.

**Figure 9:**
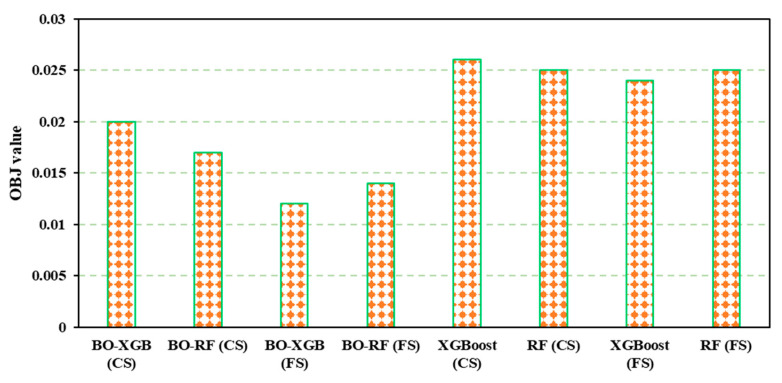
Performance analysis in terms of OBJ function.

In terms of predicting CS, from Figure 7 and Table 4, it was found that the BO-XGB model achieved an R^2^ value of 0.92, slightly lower than the BO-RF model’s R^2^ value of 0.93. However, the BO-RF model demonstrated better performance in the training dataset, with lower values of MAE, MSE, and RMSE. Again, in the test dataset, the BO-RF model continued to exhibit better performance, achieving an R^2^ value of 0.88 and an MAE of 2.55, compared to the BO-XGB model’s R^2^ value of 0.87 and MAE of 2.59. These findings suggest that the BO-RF model may be more reliable in predicting the CS of SCM than the BO-XGB model, while BO-XGB outperformed BO-RF for predicting FS. Figure 9 illustrates the objective function analysis.

**Figure 10:**
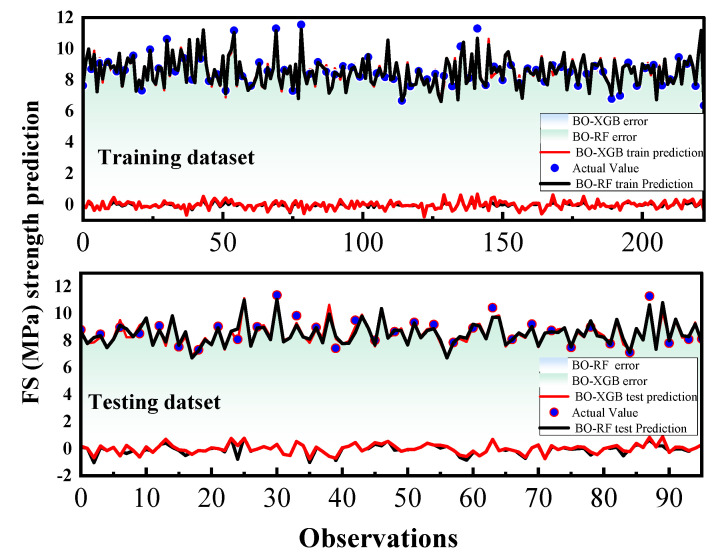
Evaluation results of FS for the training and test dataset.

**Figure 11:**
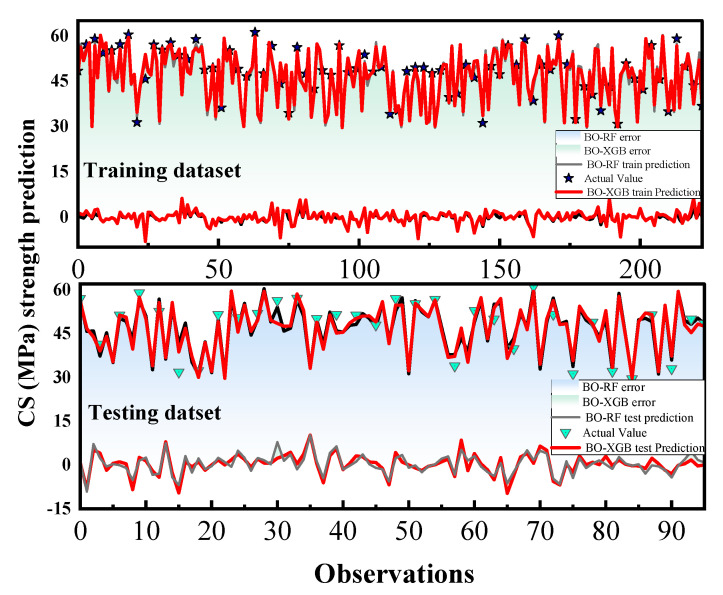
Evaluation results of CS for the training and test dataset.

The differences in performance between the BO-RF and BO-XGB models for predicting the CS and FS of SCM can be attributed to a combination of factors. BO-RF is an ensemble learning method that combines multiple decision trees, while BO-XGB is a gradient-boosting algorithm that sequentially builds a strong predictive model. These differences in modeling approaches can lead to variations in their predictive capabilities. Additionally, the SCM database features, hyperparameter settings, and the presence of potential data complexities may also play a role in the observed performance differences. In contrast, Figure 10 and Figure 11 provide a clearer representation of the adjustment profiles of the two hybrid ML models during both the training and testing stages, along with their corresponding statistical errors.

Both BO-XGB and BO-RF models displayed excellent performance in predicting the CS and FS of SCM, with MAPE values below 3%. Additionally, Figure 10 and Figure 11 show that the error ranges of both models overlap with each other, indicating that there is minimal difference between their performance in predicting shear behavior. The hybrid model’s outcome suggests that both models are capable of predicting the shear behavior of SCM with a particularly robust forecast skill.

### 5.4. Prediction Performance of Hybrid Models

#### 5.4.1. SHAP Analysis

**Figure 12:**
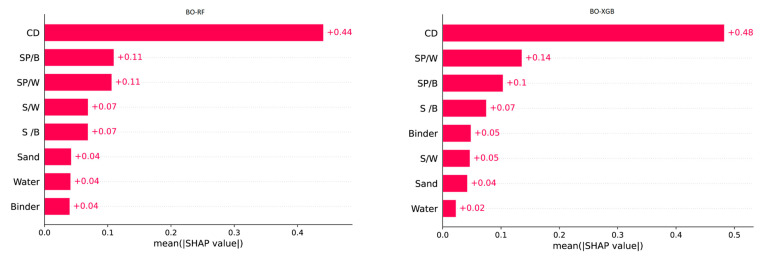
Mean SHAP values used to determine feature importance.

In general, sensitivity analysis involves exploring how a prediction is achieved through the interactions between the inputs and the outputs of a dataset. Therefore, the current study utilized the SHAP algorithm to visualize the underlying reasons behind the prediction performance of the output SCM data for selected input variables. Furthermore, the SHAP analysis enables the identification of the distinct impact of each input parameter on the prediction results. The BO-XGB model demonstrated strong predictive ability in terms of the FS of SCM, while the BO-RF model showed better performance in predicting CS (Table 3 and Figure 7). As a result, these two models were considered appropriate for the SHAP analysis. It is important to mention that the selection of these models was based on their individual strengths in predicting CS or FS and their ability to identify feature importance ranking. Input parameters with larger SHAP absolute values are deemed more impactful. In this current study, the SHAP values of all input parameters are generated through BO-XGB and BO-RF models, as depicted in Figure 12.

In this study, the SHAP magnitudes are ordered from the largest to the smallest values, which is allowed for an assessment of the relative importance of every input feature for predicting CS and FS. Although the two hybrid models exhibit different mean SHAP values, they presented similar rankings for the contribution of every input parameter. The results shown in Figure 12 indicate that the curing days (CD) of the material is the most influential input feature in predicting both CS and FS. This suggests that the curing period is the most crucial factor that affects the overall strength of the SCM.

**Figure 13:**
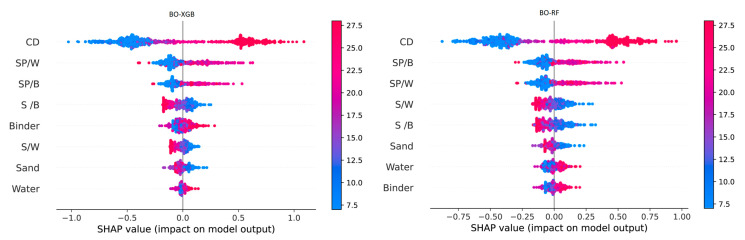
Visualization of SHAP impact values on model outputs.

The results of the SHAP analysis are consistent with established practical knowledge that the strength properties of SCM are enhanced with increased hydration ages [80,81]. According to the results, SP/B is the second most influential input in the BO-RF model, while SP/W is the second most influential input in the BO-XGB model. Interestingly, both models exhibited congruent rankings for these two influential features, occupying the second and third positions, respectively. Additionally, The S/W and S/B ratios are found to be important parameters for predicting the CS and FS of SCM. The BO-RF model ranked them as the fourth and fifth most significant factors, respectively, with similar mean SHAP values. However, the BO-XGB model displayed an opposite trend, with the S/B ratio ranked higher in importance than the S/W ratio. The strength of SCM mainly depends on the proper mix proportions of sand, binder and water-to-binder ratio [82]. The strength properties of SCM specimens rely on the rapid dissolution of mixing raw materials, which requires careful attention to the water content during the mixing process [83]. Figure 13 also indicates that the contribution of sand, water, and binder to the model outputs was minor, possibly due to a lack of variety of these parameters in the employed data set. This research used three sets of sand, water, and binder to produce SCM, which might be less variable. The influence of sand, water, and binder is expected to be quantified with higher accuracy if their mass combination could be more diverse.

The SHAP summary plot for the BO-XGB and BO-RF models is presented in Figure 13, with the input variable ranking order being consistent with the results presented in Figure 12. The models’ outcomes are presented using a violin plot, where the warm colors signify the variation in a particular value across the input features. The color spectrum ranges from blue to red, corresponding to values from low to high. It is essential to observe that a red-colored dot represents a feature value that corresponds to a high SHAP value, indicating its significance in the model’s output. For example, CD is the most influential feature selected by both models; the lower x-axis represents early days with lower strength, while the upper x-axis corresponds to higher days with higher strength. When a feature appears blue in the lower x-axis and higher in the right side of the upper x-axis in a mean shape summary plot, it indicates that the feature has a varying influence on the response variable across different levels of the feature. Therefore, if the feature appears blue on the lower x-axis and higher on the upper x-axis, it implies that the feature negatively impacts the response variable at lower levels but positively impacts the response variable at higher levels. The influence of curing days on the strength of SCM is low at the beginning, but high over time increases. This result is supported by the experimental findings shown in Figure 4 and was also explained in our previous work [84]. As the water–binder ratio is fixed in a constant value at 0.5, other features such as SP/W, SP/B, S/W, and S/B appear symmetrical in Figure 13. The SP/W and SP/B value rises along with the SHAP value increase, while the S/W and S/B value decreases as SHAP value decreases. Other parameter values vary according to the respective model.

#### 5.4.2. Permutation Importance

Permutation importance (PI) evaluates the importance of each input feature in a machine learning model. Table 5 compared the influence of each feature on the output parameter based on the PI score for BO-XGB. The CD has the highest importance, with a mean value of 0.621 and a standard deviation of 0.049. The CD was found to have approximately 5 times more influence on predicting CS and FS over SP/W. SP/W and SP/B have the second and third-highest importance, respectively, with mean values of 0.125 and 0.116 and standard deviations of 0.009 and 0.010. The permutation importance score of SP/W is higher than SP/B, indicating SP/W has a more significant prediction of the strength of SCM than SP/B. The remaining features (binder, water, S/B, S/W, and sand) had lower importance values, with mean values ranging from 0.015 to 0.006, and standard deviations fixed at 0.001. These results suggest that the CD, SP/W, and SP/B features are the dominant factor in predicting the strength of SCM, while the other features have a lesser impact on the model’s performance.

Table 6 detailed the PI result for BO-RF. The CD has the highest permutation importance score, 0.745, followed by SP/B, with a score of 0.335, and SP/W, with a score of 0.240. The remaining features have relatively lower scores, indicating they have less importance in predicting the outcome variable.

#### 5.4.3. Leave One Out Importance

The LOO method can show which data points have a large impact on the model’s performance and help with model selection and evaluation. Table 7 compares the input parameter based on LOO score. The influential parameter ranking trend is similar to the PI importance score and the mean Shapley value score visualized in Figure 12. The CD has the highest leave-one-out importance value, 0.632, which indicates that it has the most significant impact on the model’s performance in predicting SCM strength. On the other hand, the features sand and S/W have the lowest leave-one-out importance values, 0.023 and 0.024, respectively, indicating that they have the least impact on the model’s performance. The results suggest that CD is the most critical feature for predicting the output parameter in this model, followed by SP/W and SP/B.

Table 8 detailed the LOO result for BO-RF. The results demonstrated that the CD parameter has the highest importance value of 0.739, followed by SP/B and SP/W, with scores of 0.506 and 0.498, respectively. The remaining parameters, including S/W, S/B, water, sand, and binder, have relatively lower importance scores.

### 5.5. Model Efficiency

For predicting CS and FS, the accuracy did not change significantly. Therefore, we have used FS as an output parameter to evaluate the traditional ML model efficiency. Traditional statistical approaches were designed based on the idea that comprehending the fundamental mechanisms is enough to make predictions about future results. These methods operate under the assumption that data adheres to a stochastic model, which is a means of estimating probability distributions by accommodating random fluctuations. The traditional statistical modelling approach originates from a community that emphasizes the importance of unravelling mysteries to gain deeper insights into the underlying straightforward natural phenomena. 

Moreover, the methods used in this study are based on ML models. ML techniques assume that the black box is intricate and unfamiliar, which is distinct from the earlier assumption of simple stochastic processes. This dissimilar assumption has great significance and has led to numerous debates within the scientific community. Finally, the authors analyzed the data set of SCM materials by different ML models and found better prediction performances of hybrid models. This comparison, displayed in Table 9, depicted that hybrid models, especially the BO-RF and BO-XGB models, presented better prediction advantages over the employed traditional models. The computational efficiency of adopted and traditional models has also been provided.

**Figure 14:**
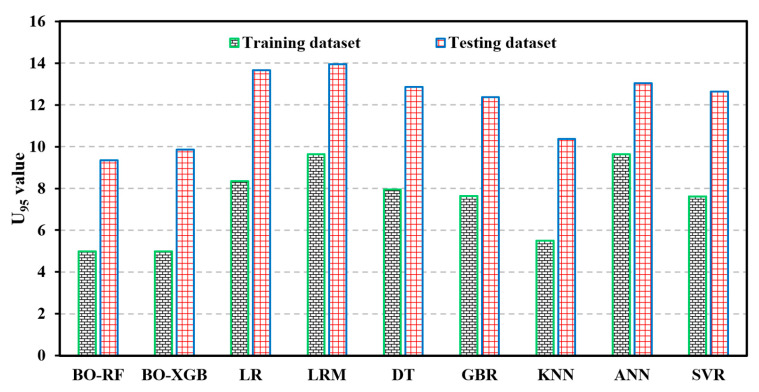
Evaluation results of uncertainty analysis test.

Figure 14 illustrates the uncertainty analysis test result, and it can be seen that both hybrid ML models exhibit the lowest amount of uncertainty compared to the traditional models.

This finding highlights the enhanced predictive capability and robustness of the hybrid model in handling and reducing uncertainty in the predictions.

## 6. Conclusions

The primary objective of this study was to use ML soft computing methods to forecast the strength behavior of SCM. Based on our experimental data, two hybrid machine learning models were developed to predict the CS and FS of SCM. The experimental specimens conducted in this study provided 320 data points for analysis, and these data points were split into training and testing sets, with 70% used for model training and the remaining 30% reserved for testing purposes. 

The study utilized a Bayesian hyperparameter optimization method for tuning the hyperparameters of the ML algorithms. This approach aimed to enhance the predictive capability of the proposed model and surpass the performance of prior transformation models in capturing the intricate relationships between the SCM strength and different input parameters. Furthermore, we employed the SHAP algorithm, permutation importance and leave-one-out importance to assess the comparative importance and ranking of the input variables with respect to the output variables CS and FS. In conclusion, the results of this study can be summarized by highlighting the following key points.

1. This study employed an experimental approach to investigate the strength behavior of SCM. Two Bayesian-optimized machine learning models were used to predict the compressive strength (CS) and flexural strength (FS) of SCM, and exhibited good performance in both training and testing phases with a high R^2^ value (>0.91) and low error. Furthermore, the MSE, and RMSE values confirmed the efficiency of the recommended BO-XGB and BO-RF models with minimal error.
2. Through Bayesian optimization, the BO-XGB and BO-RF models in this study showed impressive prediction efficiency, with an MAPE of at least 0.02%, indicating a significant correlation between input and output features. Additionally, the error fractions of both hybrid models were substantially reduced, confined to a narrow range of −10% to +10%.
3. BO-XGB was observed to be the most promising and efficient model for predicting FS, while BO-RF was found to be good at predicting CS with high accuracy. The optimal hyperparameters for the machine learning models were determined through 5-fold cross-validation during the training phase, which is essential for accurate predictions of unseen data and reducing overfitting problems.
4. The SHAP explanation provided by the BO-XGB and BO-RF models highlighted that the primary dominant parameter in improving the CS and FS is the curing days. However, other parameters such as SP/B and SP/W also played an essential role in predicting the outputs.
5. The SHAP summary plot of the BO-XGB and BO-RF model has been found to be a valuable tool in revealing the interdependencies between input and output parameters, providing insights into the SCM strength prediction model and identifying the key factors that governed the prediction accuracy. These findings provide valuable insight into the important factors that contribute to the CS and FS of SCM and can be utilized in the development of more accurate predictive models in future research.
6. The combination of permutation and leave-one-out importance techniques offers a comprehensive and reliable framework for conducting sensitivity analysis and evaluating the necessity of various features in a model. This approach can be particularly useful in understanding the factors that influence the strength of SCM. This study demonstrated that the Shapley value, permutation importance, and leave-one-out importance methods produced consistent rankings of feature importance for predicting the strength of SCM. CD and SP/W were the most influential parameters for predicting SCM strength.
7. The present research acquired the dataset from controlled experimental conditions, which were meticulously conducted under strict laboratory settings. The conditions were cautiously monitored and recorded to ensure that the experimental outcomes were not influenced by external factors that may compromise the validity and reliability of the results. This rigorous approach ensured that the data used in this study is reliable, and the proposed hybrid models are very capable of predicting the CS and FS properties of SCM composites, the results of which might apply in the construction industry.

## Figures and Tables

**Table 1:** Experimental design of SCM specimens.

Sand/Binder	Superplasticizer/Binder	Curing Days
1.5	0	7 or 28 days
	0.10%	
	0.13%	
	0.20%	
2.0	0	7 or 28 days
	0.10%	
	0.11%	
	0.12%	
	0.13%	
	0.14%	
	0.17%	
	0.20%	
	0.23%	
3.0	0	7 or 28 days

**Table 2:** A descriptive evaluation of statistical parameters of the dataset.

Parameters	Unit	Average	Standard	Minimum	Max	Operation
Binder	gm	194.72	30.92	150	240	Input
Sand	gm	405.27	30.92	360	450	Input
Water	gm	97.36	15.46	75	120	Input
CD	Day	16.87	10.21	7	28	Input
S/B	gm	2.17	0.53	1.5	3	Input
S/W	gm	4.34	1.06	3.64	6	Input
SP/W	gm	0.00033	0.00038	0	0.0011	Input
SP/B	gm	564.68	12.63	533.04	594.81	Input
Mass changes during CD	gm	40.24	0.47	37.78	41.29	Input
Flexural strength (MPa)	MPa	40.04	0.054	39.93	40.21	Output
Compressive strength (MPa)	MPa	572.00	12.92	540.69	601.11	Output

**Table 3:** Optimized hyperparameters of hybrid ML model.

Models	No	Name	Range	Optimal Parameter		Purpose
				FS	CS	
BO-XGB	1	Number of estimators	50–500	118	140	Specify number of trees
	2	Learning rate	0.01–1	0.11	0.11	Controlling step size
	3	Maximum depth	3–10	6	8	Controlling tree depth
	4	Min child weight	1–10	3	5	Sample threshold
	5	Sub sample	0.5–1	0.77	0.83	Train data sampling
	6	Reg alpha	(1 × 10^−9^)–1	1 × 10^−9^	1 × 10^−7^	L1 regularization
	7	Reg lambda	(1 × 10^−9^)–1	0.005	0.00	L2 regularization
	8	Gamma	0–1	0.918	0.918	Minimum loss reduction
	9	Colsample by tree	0.5–1	0.722	0.646	Column subsampling ratio
BO-RF	1	Number of estimators	50–500	17	17	Specify number of trees
	2	Maximum depth	2–20	18	17	Controlling tree depth
	3	Minimal gain for performing split	2–10	2	2	minimum samples required to split a node
	4	Minimum sample for leaf	1–10	1	1	Minimum samples required in a leaf node.
	5	Maximum number of features	“sqrt”, “log2”, “None”	“sqrt”	“None”	Maximum number of features considered for each split

**Table 4:** Performance evaluation of the ML model.

Model		Training-70%					Testing-30%				
ML	Strength	R^2^	MAE	MSE	RMSE	MAPE	R^2^	MAE	MSE	RMSE	MAPE
BO-XGB	CS	0.92	1.48	4.49	2.12	0.01	0.87	2.59	11.48	3.38	0.03
BO-RF	CS	0.93	0.97	1.96	1.40	0.02	0.88	2.55	12.68	3.56	0.03
BO-XGB	FS	0.96	0.09	0.01	0.13	0.02	0.91	0.26	0.13	0.37	0.02
BO-RF	FS	0.95	0.09	0.01	0.13	0.01	0.90	0.27	0.13	0.37	0.03
XGBoost	CS	0.98	0.73	1.03	1.01	0.02	0.78	2.80	15.66	3.95	0.06
RF	CS	0.97	0.98	2.04	1.42	0.03	0.81	2.66	13.89	3.72	0.06
XGBoost	FS	0.99	0.01	0.001	0.03	0	0.81	0.35	0.20	0.44	0.04
RF	FS	0.97	0.11	0.02	0.15	0.01	0.80	0.31	0.16	0.15	0.04

**Table 5:** PI score for each feature of SCM for BO-XGB.

Features Name	Mean	Standard Deviation
CD	0.621	0.049
SP/W	0.125	0.009
SP/B	0.116	0.010
Binder	0.015	0.001
Water	0.011	0.001
S/B	0.010	0.001
S/W	0.007	0.001
Sand	0.006	0.001

**Table 6:** PI score for each feature of SCM for BO-RF.

Features Name	Mean	Standard Deviation
CD	0.745	0.15
SP/B	0.0.335	0.075
SP/W	0.240	0.040
S/W	0.015	0.001
S/B	0.011	0.001
Water	0.010	0.001
Sand	0.008	0.001
Binder	0.003	0.001

**Table 7:** LOO importance score each feature of SCM for BO-XGB.

Feature	Leave-One-Out Importance
CD	0.632
SP/W	0.142
SP/B	0.129
Binder	0.032
Water	0.030
S/B	0.026
Sand	0.024
S/W	0.023

**Table 8:** LOO importance score each feature of SCM BO-RF.

Feature	Leave-One-Out Importance
CD	0.739
SP/B	0.506
SP/W	0.498
S/W	0.195
S/B	0.025
Water	0.018
Sand	0.015
Binder	0.011

**Table 9:** Efficiency measurement of employed HML models over the traditional models.

Traditional Models and Adopted Models	Training Dataset				Testing Dataset				Time (s)
	R^2^	RMSE	VAF	RRSE	R^2^	RMSE	VAF	RRSE	-
Linear Regression	0.58	0.64	0.58	0.62	0.56	0.68	0.56	0.58	5.8
Lasso regression model	0.54	0.66	0.54	0.62	52	0.72	0.52	0.63	37.6
Decision Tree Regressor	0.71	0.47	0.71	0.45	0.70	0.51	0.70	0.41	2.3
Gradient Boosting Regressor	0.77	9.24	0.77	0.17	0.76	9.70	0.76	0.38	7.4
KNN Regression	0.86	0.22	0.86	0.35	0.83	0.40	0.83	0.38	3.7
ANN	0.54	0.66	0.54	0.20	0.52	0.72	0.77	0.22	14
SVR	0.75	0.48	0.75	0.54	0.72	0.59	0.75	0.50	4.8
BO-XGB	0.96	0.13	0.96	0.02	0.91	0.37	0.06	0.02	54
BO-RF	0.95	0.13	0.95	0.04	0.90	0.37	0.06	0.04	58

## Data Availability

The dataset used in this study is available upon request from the corresponding author.

## Abbreviations

SCM	Self-compacting mortar
XGBoost	Extreme gradient boosting
RF	Random Forest
BO-XGB	Baysian-optimized Extreme gradient boosting method
BO-RF	Baysian-optimized Random Forest method
CS	Compressive strength
FS	Flexural strength
S/W	Sand–water ratio
S/B	Sand–binder ratio
SP/B	Superplasticizer–binder ratio
SP/W	Superplasticizer–water ratio
OOB	Out of bag
DNNR	Deep neural network regression
GEP	Genetic programming
ANN	Artificial neural network
